# Editorial: Regulation of Pollen Tube Growth

**DOI:** 10.3389/fpls.2021.658902

**Published:** 2021-02-25

**Authors:** Giampiero Cai, Delia Fernández-González, Stefano Del Duca

**Affiliations:** ^1^Department of Life Sciences, University of Siena, Siena, Italy; ^2^Institute of Atmospheric Sciences and Climate (National Research Council), Bologna, Italy; ^3^Department Biodiversity and Environmental Management, University of León, León, Spain; ^4^Department of Biological, Geological and Environmental Sciences, University of Bologna, Bologna, Italy

**Keywords:** allergic protein, vesicular trafficking, exocyst complex, pollen-pistil communication, self-incompatibility, polarized growth

In angiosperms, the pollen tube is a simple system composed of the vegetative cell and the two sperm cells which, nevertheless, accomplishes a very important process, essential for the life of flowering plants on Earth, i.e., sexual reproduction. In its simplicity, the pollen tube allowed plants to reproduce on land, even in the absence of water. Therefore, it is a very critical evolutionary factor. In the last 30 years, the pollen tube has been the object of study for many researchers around the world because of a number of reasons; apart from its biological importance, the pollen tube is a highly valuable cell model by which to analyze many aspects of plant cell biology (except for photosynthesis). The time course of cell wall deposition, the role of calcium ions in driving the apical growth of pollen tube, the action of signal transduction intermediates, the cell-cell communication, the mechanism of cell shape control by exocytosis/endocytosis are just some of the aspects on which it is possible to find articles in the scientific literature dealing with the pollen tube. Not to forget that the study of pollen tubes also has practical implications because the control of the reproductive process of plants also involves the study of genes, proteins and metabolites that promote or prevent the growth of pollen tubes. Therefore, controlling pollen tube growth can impact seed and fruit yields. Due to all these considerations, the Research Topic “Regulation of pollen tube growth” was meant to highlight some of the above aspects with updated considerations and special focus. These include contributions related to human health, pollen-pistil interaction, the growth of pollen tubes by exocytosis/endocytosis, and the rejection of the pollen tube in self-incompatibility processes.

The manuscript by Adhikari et al. is a worthwhile guide to the various processes of plant biology that find in the pollen tube a convenient way for their interpretation. The authors opted to focus their manuscript on four particularly discussed topics: the pollen tube as a cell, the dynamics of its membranes, the mechanics of its elongation, and the mechanosensory potentials involved in its elongation. The authors' critical views allow us to look at the pollen tube from a different perspective and to broaden the horizons of application of this cellular system.

Pollen contains proteins that can have effects on human health. In olive, the proteins “Ole e 1” are normally involved in the fertilization mechanism but, when pollen grains fall into the nasal mucosa, the expression of “Ole e 1” induces an allergic reaction. Examining a large period, Fernández-González et al. showed an inverse correlation between the amount of pollen produced and the quantity of the protein “Ole e 1.” This led the authors to hypothesize that “Ole e 1” synthesis is increased in years with low pollen production in order to allow for adequate pollen tube elongation. This could open up scenarios for the prevention of clinical symptomatology of pollinosis.

If we move on to how the pollen tube can grow, Zhao et al. discussed the most recent data on exocytosis and endocytosis and how they may affect the polarized growth of pollen tube. As is often the case, the authors have also introduced the new integration of mathematical modeling; indeed, the pollen tube is well-suited to a system biology approach, valuable in unlocking the puzzles of cell polarity and polar cell growth. The application of mathematically based modeling systems is very convenient to determine how a given process may affect cell shape. After a general review of vesicular trafficking, the control of the exocytosis process was the subject of the manuscript by Saccomanno et al.. The authors analyzed the involvement of phosphorylation in controlling the activity of EXO70C2, one of the subunits of the exocyst heterooctameric protein complex. Thus, a connection can be made between intracellular signaling mechanisms and the process of exocytosis. The expectation is to interpret how any extracellular signals might act on secretion of new plasma membrane and cell wall.

At a time when the pollen grain touches the stigma surface, this triggers a series of events that must lead to the activation of the pollen grain and the emission of the pollen tube. Katano et al. analyzed the effect of heat stress on changing the shape of stigma cells (the stigmatic papillae) and concluded that such a dramatic event may interfere with pollen-pistil communication. In addition to this, the authors also pointed out that the absence of pollen may also control the shape of stigma cells. This suggests how a continuous exchange of information between the male and female parts is of fundamental importance for the reproductive success of plants.

This continuous exchange of information is also fundamental for the growth and direction of the pollen tube inside the female tissue. Lora et al. focused on a few molecules in particular, including starch, arabinogalactan proteins (AGPs), and GABA in a species of commercial interest such as mango. Their results show similarities and differences with other species, supporting heterotrophic pollen tube growth at the expense of starch but not exhibiting high levels of GABA. This must always remind us of how important it is, on the one hand, to have the idea of a general cell model but, on the other hand, also to verify, on a case-by-case basis, the results that have been obtained.

As pointed out at the beginning of this Editorial, the success of the reproductive mechanism in flowering plants predicts that the pollen tube can grow undisturbed between the pistil cells. When this does not happen, it is because the plant rejects the pollen tube in a process known as self-incompatibility. Aloisi et al. have specifically analyzed this process in Citrus highlighting how in this case pollen rejection can also be dependent on the temperature at which plants are exposed. Together with the analysis of amine metabolites, these results underline once again how stable environmental conditions are of absolute importance for proper reproduction in flowering plants and how changing climatic conditions can lead to unwanted crossings.

## Author Contributions

SD, GC, and DF-G designed, selected, and organized the Research Topic with a balanced contribution between all three guest editors.

## Conflict of Interest

The authors declare that the research was conducted in the absence of any commercial or financial relationships that could be construed as a potential conflict of interest.

